# Are Daily Sedation Stops Safe in a Medical Icu?

**DOI:** 10.1186/2197-425X-3-S1-A26

**Published:** 2015-10-01

**Authors:** AS Debue, J Charpentier, M Arnaout, J Busson, C Boulila, S Cabon, J Dhumeaux, N Ericher, S Lefort, P Lucas, A Marincamp, M Reffiena, A Cariou, JP Mira, JD Chiche, Groupe de Travail sur la Ventilation

**Affiliations:** Cochin Port Royal, MICU, Paris Cedex 14, France

## Introduction

Despite evidence that daily sedation stops (DSS) by nurses reduce duration of mechanical ventilation (MV), DSS are not part of routine practice in most ICUs. Reasons for slow adoption include scepticism over efficacy, logistical burden in initiating changes, and fear of adverse events during sedation stops. Whether sedation stops are associated with significant adverse events is unknown.

## Objectives

This study aims to evaluate adverse events occurring during 2-hour DSS in a medical ICU.

## Methods

We have retrospectively reviewed charts of all pts mechanically ventilated in the 24-bed MICU of Cochin University Hospital. in the absence of contra-indication, all pts underwent a 2-hour DSS as part of our combined sedation & weaning protocols. During and at the end of DSS, adverse events, need for bolus or infusion of sedative agents, and RASS scores were prospectively recorded in a clinical information management system (Clinisoft^®^, GE Healthcare). Data are reported as median and interquartile ranges. a P < 0.05 was considered significant for all statistical tests performed.

## Results

Between 03/08 and 04/15, 9799 pts ( 5760 M/ 4039 F, 62 [46-76] y.o., SAPS2 43 [29-60], SOFA 5 [2-8]) underwent 12,474 DSS. No significant adverse event occurred in 12,217 (97.9%) of the DSS. Adverse events included patient-ventilator asynchrony (n = 159 DSS), O2 desaturation (n = 159 DSS), accidental catheter removal (n = 22), fall (n = 9). However, mild to moderate agitation occurred during 2,483 DSS (19.9%) and led to prematurely resume continuous sedation (n = 1707, 13.7%) or to administer boluses of sedative agents (n = 776, 6.2%).

**Table 1:** Sedatives administered during DSS.

	BZD	Opiates	Propofol	Others
**Infusion**	984 (57.6%)	575 (33.7%)	127 (7.5%)	21 (1.2%)
**Bolus**	182 (23.4%)	240 (30.9%)	26 (3.3%)	241 (31%)

## Conclusions

Overall, daily sedation stops are safe and associated with few adverse events in a setting with nurse-driven monitoring of neurological status. Administration of sedative agents may occasionally be needed to control mild to moderate agitation that may lead to adverse events.

